# Cross Talk between Inhibitory Immunoreceptor Tyrosine-Based Activation Motif-Signaling and Toll-Like Receptor Pathways in Macrophages and Dendritic Cells

**DOI:** 10.3389/fimmu.2017.00394

**Published:** 2017-04-07

**Authors:** Ivan Hirsch, Vaclav Janovec, Ruzena Stranska, Nathalie Bendriss-Vermare

**Affiliations:** ^1^Faculty of Science, Charles University, Prague, Czech Republic; ^2^Institute of Molecular Genetics, ASCR, Prague, Czech Republic; ^3^Institute of Organic Chemistry and Biochemistry, ASCR, Prague, Czech Republic; ^4^Cancer Research Center Marseille, INSERM U 1068, CNRS, UMR7258, Marseille, France; ^5^Institut Paoli-Calmettes, Aix-Marseille University, Marseille, France; ^6^INSERM 1052, CNRS 5286, Centre Léon Bérard, Centre de Recherche en Cancérologie de Lyon, Univ Lyon, Université Claude Bernard Lyon 1, Lyon, France

**Keywords:** plasmacytoid dendritic cell, conventional dendritic cells, macrophage, toll-like receptors, regulatory receptors, immunoreceptor tyrosine-based activation motif-associated receptor, B cell receptor-like signaling

## Abstract

The innate immune cells sense microbial infection and self-ligands by pathogen recognition receptors (PRRs), such as toll-like receptors (TLRs) and regulatory receptors (RRs), associated with immunoreceptor tyrosine-based activation motif (ITAM). Rapid activation and concerted action of PRRs signaling and feedback inhibitory mechanisms must be engaged to ensure the host defense functions and to prevent cytotoxicity associated with excessive activation. ITAM-associated RRs can generate stimulatory or, paradoxically, inhibitory signals. The network of ITAM-associated RR, together with TLR-signaling pathways, are responsible for immunogenic or tolerogenic responses of macrophages and dendritic cells to their microenvironment. In macrophages, TLR4 signaling is inhibited by low-avidity ligation of ITAM-associated receptors, while high-avidity ligation of ITAM-associated receptors results in potentiation of TLR4 signaling together with resistance to extracellular cytokine microenvironment signals. In contrast to macrophages, TLR7/9 signaling in plasmacytoid DCs (pDCs) is inhibited by high-avidity ligation of ITAM-associated RR, while low-avidity ligation does not show any effect. Surprisingly, interference of ITAM-associated receptor signaling with TLR pathways has not been reported in conventional dendritic cells. Here, we present an overview of molecular mechanisms acting at the crossroads of TLR and ITAM-signaling pathways and address the question of how the high-avidity engagement of the ITAM-associated receptors in pDCs inhibits TLR7/9 signaling. Cellular context and spatiotemporal engagement of ITAM- and TLR-signaling pathways are responsible for different outcomes of macrophage versus pDC activation. While the cross-regulation of cytokine and TLR signaling, together with antigen presentation, are the principal functions of ITAM-associated RR in macrophages, the major role of these receptors in pDCs seems to be related to inhibition of cytokine production and reestablishment of a tolerogenic state following pDC activation. Pharmacologic targeting of TLR and ITAM signaling could be an attractive new therapeutic approach for treatment of chronic infections, cancer, and autoimmune and inflammatory diseases related to pDCs.

## Introduction

Macrophages and dendritic cells (DCs) play a major role in initiating and sustaining innate and adaptive immune responses and are the nexus at which immune stimulation or suppression occurs (1–5). The innate immune cells sense microbial infection and self-ligands such as damaged or altered self, including dead cells, by pathogen recognition receptors (PRRs), such as toll-like receptors (TLRs) and lectin-like receptors (LLRs), also called C-type lectin receptors (6). Rapid activation and concerted action of PRRs signaling is needed to ensure the host defense functions after infectious challenge or tissue damage. PRR agonists and secreted cytokines and chemokines are the drivers and the major regulators of fine-tuned innate immune responses. Concomitantly, feedback inhibitory mechanisms must be engaged to prevent cytotoxicity associated with excessive activation of the innate immune cells (7). Thus, TLRs that confer functional specificity to macrophages and DC subsets trigger intracellular signaling cascades that result in the secretion of interferons (IFNs) and pro-inflammatory cytokines and activation of host defense programs necessary for innate or adaptive immune responses. The same cells also specifically express immunoreceptor tyrosine-based activation motif (ITAM)-associated receptors that can modulate TLR-signaling pathways (3, 8, 9). The conserved ITAM-signaling motif, with a consensus sequence YXXL/I–X_6–8_–YXXL/I (where X denotes any amino acid), is present in the cytoplasmic tail of transmembrane adaptor molecules associated with multiple receptors. Initially discovered ITAM-associated receptors, including the T-cell receptor, B-cell receptor (BCR), and Fc receptors (FcRs), were shown to induce phosphorylation of the tyrosines within the ITAMs, to recruit Syk tyrosine kinases, and to activate the immune cell. More recent studies have shown that some ITAM-associated receptors mainly in the innate immune cells efficiently inhibit downstream signaling triggered by other types of PRRs.

Here, we present an overview of molecular mechanisms acting at the crossroads of TLRs and regulatory receptors (RRs) signaling and address the question of how the engagement of the ITAM-associated receptors in macrophages and two subtypes of DCs, conventional dendritic cells (cDCs) and plasmacytoid DCs (pDCs), inhibits cytokine and TLR7/9 signaling. We compare ITAM-mediated inhibitory mechanisms and function of the ITAM-associated receptors in these cell types. We focused our review on the neglected observation that TLR signaling in pDCs is inhibited by high-avidity engagement of the ITAM-associated RRs; while in macrophages, it is inhibited by low-avidity engagement of these receptors.

On the basis of this comparison, we assess the functions of the ITAM-associated receptors in those cells types. We hypothesize that while antigen presentation and cross-regulation of cytokine and TLR signaling are the principal functions of ITAM-associated receptors in macrophages, the major role of these receptors in pDCs is the inhibition of cytokine production and reestablishment of a tolerogenic state following pDC activation.

## Immunogenic and Tolerogenic Receptors of DCs

**Figure 1:**
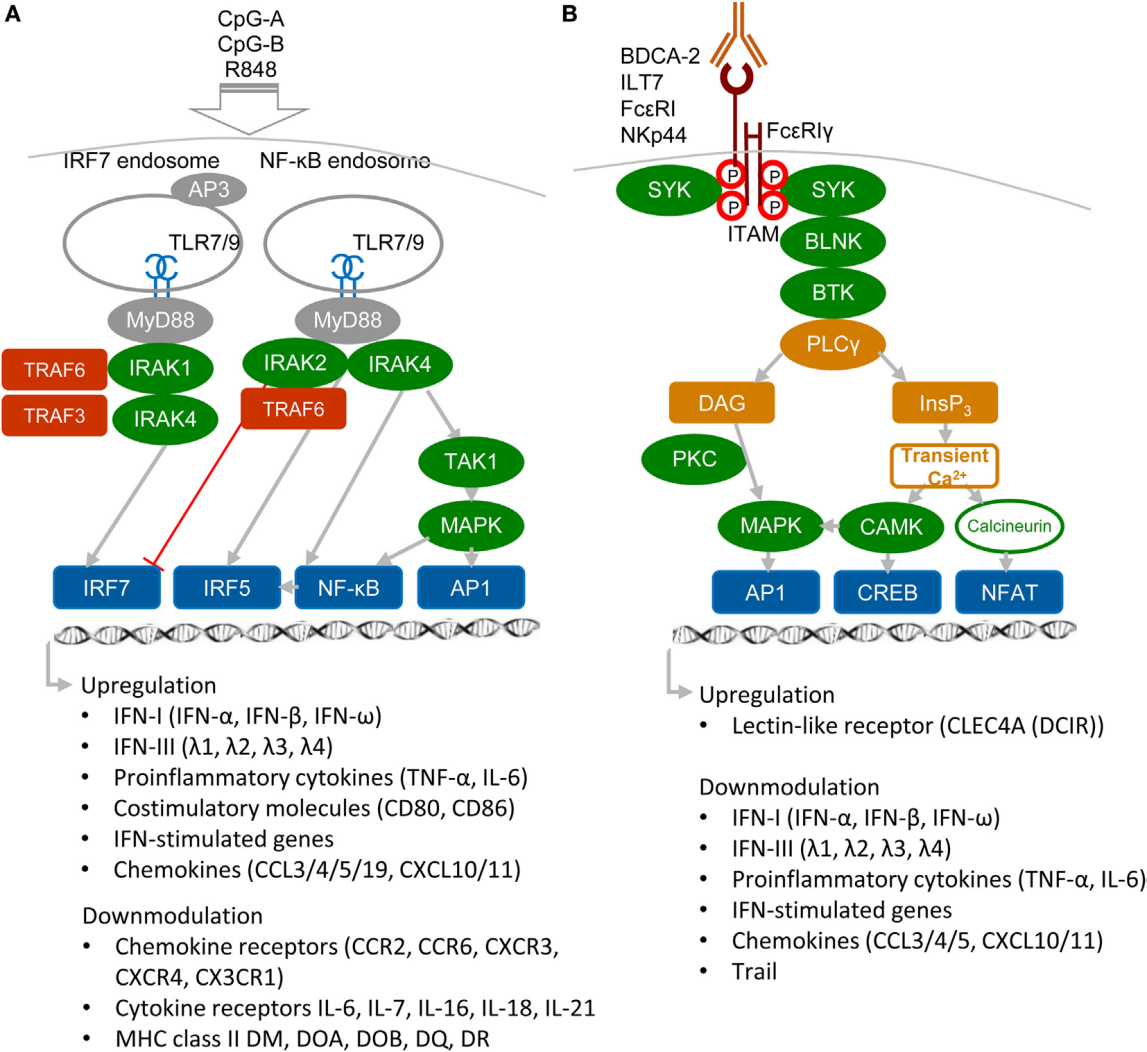
**Activatory signaling pathways control TLR7/9 signaling in plasmacytoid DCs (pDCs) (simplified view)**. **(A)** Ligation of TLR7 or TLR9 with their agonists, CpG-A or CpG-B, induces different types of signaling. Aggregating CpG-A is transported in the presence of adaptor protein 3 (AP3) to the interferon-regulatory factor 7 (IRF7) endosomes, where a signalosome composed of IRAK1, IRAK4, TRAF3, and TRAF6 is formed. IRF7 signalosome activates production of interferons (IFNs)-I (IFN-α/β) and IFN-III (IFN-λ). In contrast, transcriptome studies showed that CpG-A downmodulates expression of some chemokine and cytokine receptors and MHC class II molecules (16). Monomeric CpG-B is transferred to the NF-κB endosomes, where a signalosome is formed from the set including IRAK4, TRAF6, transforming growth factor β-activated kinase 1 (TAK1), and NF-κB/MAPK/IRF5, which leads to maturation of pDCs and formation of pro-inflammatory cytokines and chemokines. IRAK2 suppresses IFN-I/III production but positively controls production of pro-inflammatory cytokines (3, 5, 16). **(B)** Regulatory signaling pathways. Crosslinking of regulatory receptors, such as blood dendritic cell antigen 2 (BDCA-2), ILT-7, FcεRI, or NKp44, induces activation signals that lead to phosphorylation of tyrosines (shown by red circles) within the FcεRIγ immunoreceptor tyrosine-based activation motif (ITAM) and to recruitment of spleen tyrosine kinase (SYK) kinase. FcεRIγ-mediated B-cell receptor (BCR)-like signaling involving SYK, B-cell linker protein (BLNK), Bruton’s tyrosine kinase (BTK), and PLCγ results in hydrolysis of phosphatidylinositol 4,5-biphosphate to diacylglycerol (DAG) and inositol-1,4,5-triphosphate (InsP_3_). DAG activates protein kinase C (PKC) and mitogen-activated protein kinase (MAPK), which contribute to activation of activator protein (AP1) and NF-κB. InsP_3_ leads to transient release of intracellular Ca^2+^ stores followed by activation of calmodulin-dependent kinase (CAMK) and cyclic-AMP-responsive-element-binding protein. In parallel, calcineurin contributes to activation of the nuclear factor of activated T cells (NFAT). Transcriptome studies showed that BDCA-2 downmodulates expression of IFN-I, IFN-III, some chemokines and cytokines, and IFN-stimulated genes (16).

Plasmacytoid DCs are a highly specialized subset of DCs that function as sentinels for viral infection and cancer. They are responsible for production of type I and III IFNs, IFN-I (namely IFN-α, β, and ω) and IFN-III (IFN-λ1, λ2, λ3, and λ4 also called IL-29, IL-28A, IL-28B, and IL-28C), pro-inflammatory cytokines, and antigen presentation (Figure 1A). pDCs are able to detect genetic material of viruses with a subset of nucleotide-sensing TLRs localized in the endosomal compartment: TLR7, which recognizes single-stranded RNA, and TLR9, which recognizes DNA. TLR7 also recognizes synthetic imidazoquinoline components, for example Resiquimod (R848), whereas TLR9 recognizes synthetic CpG oligonucleotides. Ligation of TLR9 with aggregating CpG-A oligonucleotides in the early endosomes triggers the adaptor protein 3-dependent MyD88-IRF7 pathway that includes TLR adaptor MyD88, interleukin-1 receptor-associated kinase 1/4 (IRAK1/4), tumor necrosis factor receptor-associated factors 3 and 6 (TRAF3/6), and interferon-regulatory factor 7 (IRF7), and that results in the type I IFN production (3, 5, 10, 11) (Figure 1A). Activated IRF7, which is constitutively expressed in pDCs, translocates to the nucleus and, together with ATF-2, c-Jun, and nuclear factor kappa B (NF-κB) subunits p50 and RelA, initiates the transcription of IFN-I (12). Furthermore, it has been demonstrated that TLR9-mediated induction of transforming growth factor β-activated kinase 1 (TAK1) and of inhibitor of nuclear factor κB kinase subunit β, followed by the IFN-β-stimulated activation of the JAK-STAT1/2 pathway, are essential for production of IFN-α (13). This second loop of IFN-I signaling induced by IFN-β secreted by pDCs triggers a robust IFN-I/III response and expression of IFN-stimulated genes, and it can be blocked by mAbs against secreted IFN-I or IFN-α/β receptor. In contrast to IRF7-mediated production of IFN-I, monomeric CpG-B oligonucletides are transferred to an endolysosomal compartment where they activate the MyD88–NF-κB pathway that triggers expression of mitogen-activated protein kinases (MAPKs) and IRF5 (14, 15) (Figure 1A). Both, NF-κB and MAPKs, stimulate secretion of chemokines and of the pro-inflammatory cytokines interleukin-6 and tumor necrosis factor-α (TNF-α) and stimulate expression of co-stimulatory molecules, such as CD80 (B7.1) and CD86 (B7.2).

In addition to nucleotide-sensing TLRs, pDCs also recognize pathogens through a battery of cell surface RRs, including FcRs and LLRs. The principal function of these RRs on pDCs is to facilitate antigen capture and presentation and to prevent aberrant immune responses by modulating production of IFN-I and pro-inflammatory cytokines (3, 5, 11) (Figure 1B). RRs deliver their signal through immunoreceptor tyrosine-based inhibition motif (ITIM) or through ITAM-associated adaptors, like the γ-chain of FcεRIγ or DNAX activation protein 12 (DAP12). Among ITAM-signaling receptors, blood dendritic cell antigen 2 (BDCA-2, CD303, CLEC4C) (17, 18), immunoglobulin-like transcript (ILT7, CD85g) (19, 20), and FcεRI (21) signal through FcεRIγ, while NKp44 (22) and mouse pDC–specific Siglec-H (23) signal through DAP12. In pDCs, triggering of these receptors initiates a signaling pathway involving spleen tyrosine kinase (SYK), Bruton’s tyrosine kinase, B-cell linker protein, phospholipase Cγ 2 (PLCγ2), MEK-ERK, and induction of intracellular Ca^2+^ mobilization, similar to the pathway that occurs downstream of the BCR (18). Despite the similarity with BCR pathway, BDCA-2 signaling does not lead to the activation of the canonical NF-ĸB pathway monitored by the IĸBα (16). Other RRs of pDC, such as dendritic cell immunoreceptor (DCIR, CLEC4A) (24), contain an ITIM motif. In spite of differences in ITAM or ITIM motifs, all these receptors inhibit TLR7/9 signaling (17, 18). Thus, the production of IFN-I in pDCs is controlled positively by immunogenic TLR7/9 and negatively by tolerogenic RRs.

Human cDCs, also called classical or myeloid dendritic cells, can be divided into at least two subsets. The more common mDC1s (BDCA-1^+^CD1c^+^), which produce inflammatory cytokines and chemokines, are major stimulators of T cells (25). The second subset, extremely rare mDC2s (BDCA-3^+^ XCR1^+^Clec9A^+^) produce IL-12 and cross-present antigens for CD8 class I-restricted cytotoxic T lymphocytes (26, 27). cDCs detect invading microbes with the cell surface-expressed TLR1 and TLR2, which recognize peptidoglycan and lipoproteins, and endosomal compartment-localized TLR3, which recognizes double-stranded (ds)RNA, and TLR8, which recognizes single-stranded RNA (3–5). mDC2s are major producers of IFN-III, induced *via* dsRNA-sensing TLR3 pathway, independent of TLR7 (27). Immature cDCs sample the surrounding microenvironment for pathogens by numerous PRRs, including TLRs and ITAM-associated LLRs (6, 28, 29). Among ITAM-associated LLR expressed on cDCs, Dectin 2 (CLEC6A) and macrophage-inducible C-type lectin (Mincle, CLEC4E) associate with the ITAM-containing adaptor FcRγ, while myeloid DAP12-associated lectin-1 associates with the adaptor DAP12. Ligand binding to these LLRs leads in cDCs to phosphorylation of ITAM and recruitment of SYK like in pDCs (Figure 1B). However, in contrast to pDCs, recruitment of SYK in cDCs is followed by the formation of SYK–CARD9–BCL9–MALT1 complex, activation of the NF-ĸB subunit c-Rel, and production of pro-inflammatory cytokines (4, 6, 30). Surprisingly, this signaling pathway can result in the IRF5-mediated production of IFN-β without engagement of TLRs (31). Previous report of an alternative mechanism based on recognition of fungal infection by TLR7, independently of Dectin-1, makes induction of IFN-β in cDCs the matter of debate (32). While ligation of ITIM-associated LLR, such as myeloid C-type lectin-like receptor (MICL) or DCIR in cDCs inhibits TLR4 and TLR8 signaling (24, 33, 34), suppression of TLR signaling by ligation of ITAM-associated LLRs in cDCs has not been reported (28). These results together with the recent observation showing that Dectin-1-activated pDCs promote Th2-type T cell responses while Dectin-1-activated cDCs do the opposite, point to the importance of combination of PAMP, PRR, and the cell context in the regulation of adaptive immune responses by innate immunity (4, 35).

## Negative Signaling by Itam-Associated Receptors in Macrophages

**Figure 2:**
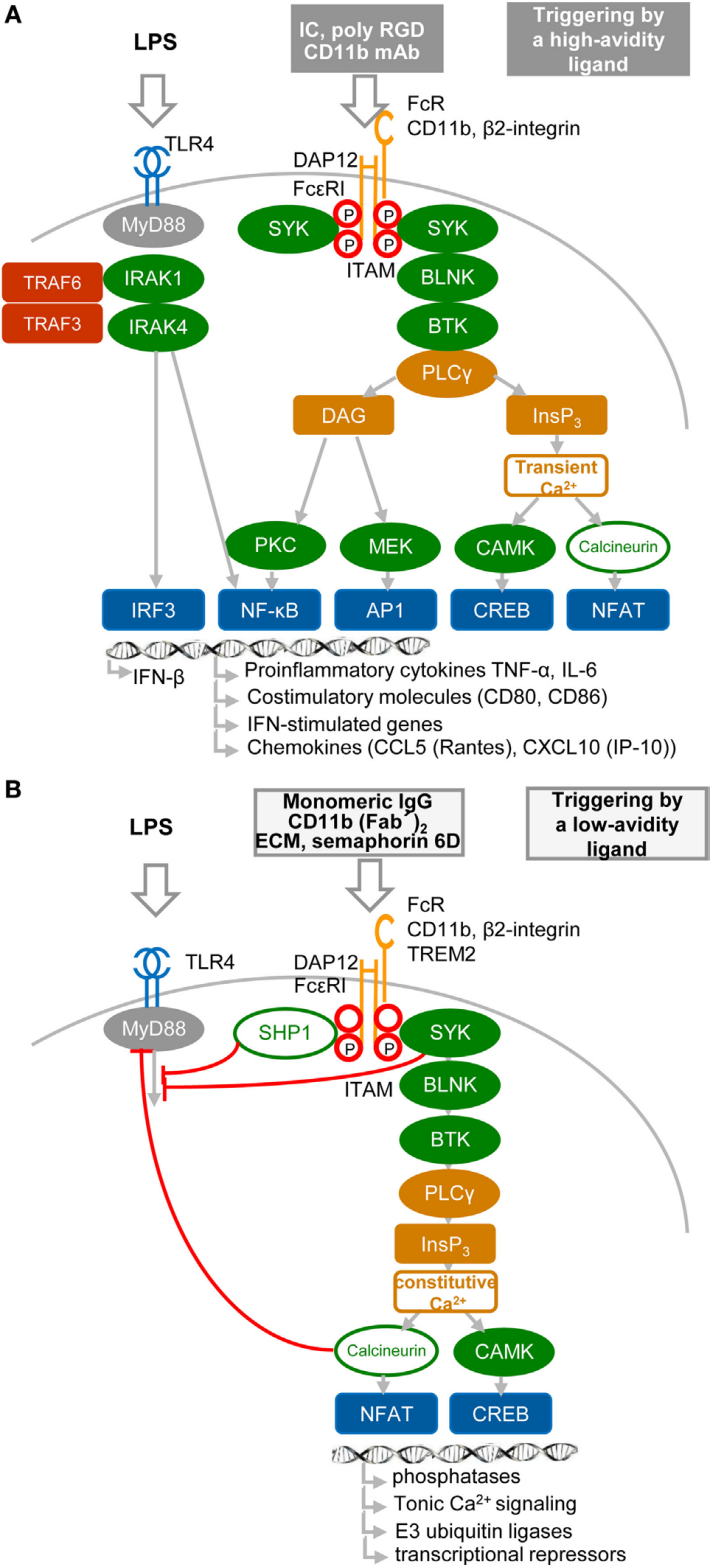
**High- and low-avidity engagement of the immunoreceptor tyrosine-based activation motif (ITAM)-associated receptors in macrophages**. **(A)** High-avidity ligation of the ITAM-associated receptor results in synergy of ITAM and TLR4 signaling. High-avidity ligation of macrophage receptors, such as Fc receptors (FcRs) or β2-integrin [e.g., by cross-linking of FcR with immune complexes (IC), or β2-integrin with CD11b mAb or poly arginine–glycyl–aspartic acid (RGD) motifs (8)], induces activation signals that lead to phosphorylation of tyrosine residues within the DNAX activation protein 12 (DAP12) ITAM motif and to recruitment of spleen tyrosine kinase (SYK) kinase. DAP12 ITAM-mediated signaling involving SYK, B-cell linker protein (BLNK), Bruton’s tyrosine kinase (BTK), and PLCγ proceeds as the signaling triggered by regulatory receptors in plasmacytoid DCs (Figure 1B). Ligation of TLR4 with its agonist (LPS) induces formation of a signalosome (from a set including IRAK1, IRAK4, TRAF3, and TRAF6), which activates IRF3 and production of IFN-β and pro-inflammatory cytokines (8, 9, 46). Tyrosine residues in ITAM motifs are shown by red circles. **(B)** Low-avidity ligation of the ITAM-associated receptor [e.g., by ligation of FcγR with monomeric IgG as exemplified by ligation of FcγRIIA with AT-10 F(ab′)_2_ (44), β2-integrin with CD11b F(ab′)_2_, or with Extracellular matrix (ECM) or triggering receptor expressed on myeloid cells 2 (TREM2) with semaphorin 6D] results in inhibition of TLR4 signaling. Low-avidity engagement of a high-affinity receptor results in recruitment of the SRC-homology-2 (SH2)-domain-containing protein tyrosine phosphatase 1 (SHP1), SHP2, and SH2-domain-containing inositol-5-phosphatase (SHIP) to the monophosphorylated membrane-distal tyrosine (Y304) of ITAM (44), shown by a letter P within a red circle. SHP1, SHP2, or SHIP can dephosphorylate TLR4 signaling intermediates. Low-avidity receptor ligation changes the balance between calcium and protein kinase C (PKC)-mediated pathways, leading to increased activity of calmodulin-dependent kinase (CAMK) and nuclear factor of activated T cells (NFAT) in the absence of NF-κB or mitogen-activated protein kinase activation.

Results obtained during the two last decennia show that immune receptors associated with an ITAM can generate stimulatory or, paradoxically, inhibitory signals (36–44) (Figures 2A,B). These findings, obtained mostly in macrophages, provoked intense research into underlying mechanisms, as well as semantic debate (45). Inhibition can be readily explained by the paired co-clustering of ITIM-bearing receptors with the targeted ITAM-associated receptor, which brings them into close proximity for the consecutive inhibitory action of the tyrosine phosphatase SRC-homology-2 (SH2)-domain-containing protein tyrosine phosphatase 1 (SHP1) and SH2-domain-containing inositol phosphatase-1 (SHIP) (9, 24, 34, 42, 44) (Figure 2B). The work of several laboratories suggests that positive or negative control of immune responses, in the case of ITAM alone, is determined by avidity of ITAM-associated receptors to their ligands. The resulting “signal-switch hypothesis” is based on the observation that cross-linking by multimeric or high-avidity engagement of the ITAM-associated receptors leads to complete phosphorylation of ITAM tyrosine residues followed by the recruitment of SYK and to cell activation that synergizes IFN-I production, but inhibits cytokine signaling (8, 9, 39, 45, 46) (Figure 2A). In contrast, monovalent or low-avidity engagement of the ITAM-associated receptor results in monophosphorylation of the membrane-distal tyrosine (Y304) of ITAM allowing a transient recruitment and minimal activation of Syk (44) followed by actin depolymerization and translocation of protein or lipid phosphatases (SHP1, SHIP) instead of SYK to the ITAM in lipid rafts. Tyrosine phosphatases SHP1/2 and lipid phosphatase SHIP recruited to partially phosphorylated ITAM can inhibit TLR4 signaling by dephosphorylation of signaling intermediates, but concomitantly cell sensitivity to extracellular cytokines increases (Figure 2B). If a high-avidity stimulation of other receptors, such as FcγRs, FcεRI, the tumor necrosis factor receptors, chemokine CC-motif receptor 2, or TLRs, occurs in the proximity of a weak-avidity stimulation, the high-avidity-stimulated receptor is recruited toward the inhibitory SHP1 (9, 38, 42, 43, 46, 47). High-avidity signaling is deactivated by SHP1 in rafts and completed after internalization and segregation into polarized clusters called “inhibisomes,” with SYK present at their periphery (9, 42).

## Mechanisms Inhibiting TLR Signaling in Macrophages

Spatiotemporal compartmentalization of inhibitory ITAM-containing receptors into lipid rafts is a key event in the triggering of several ITAM-mediated inhibitory signals. Thus, the presence of ITAMs in inhibisome rafts can be responsible for induction of a phosphatidylinositol 3-OH kinase (PI3K)- and PLCγ2-mediated imbalance characterized by accumulation of inositol-1,4,5-triphosphate (InsP_3_) and low levels of diacylglycerol (Figure 2B). This imbalance results in triggering of constitutive calcium and MAPK signaling without phosphorylation of IκB at position Ser32 (47) and without activation of NF-κB, but it is sufficient to activate nuclear factor of activated T cells (8, 48). The importance of calcium signaling is highlighted by the finding that release of intracellular calcium activates the calcium-dependent phosphatase calcineurin, which is involved in the inhibition of TLR signaling by targeting the adaptor proteins MyD88 and TRIF (47).

**Figure 3:**
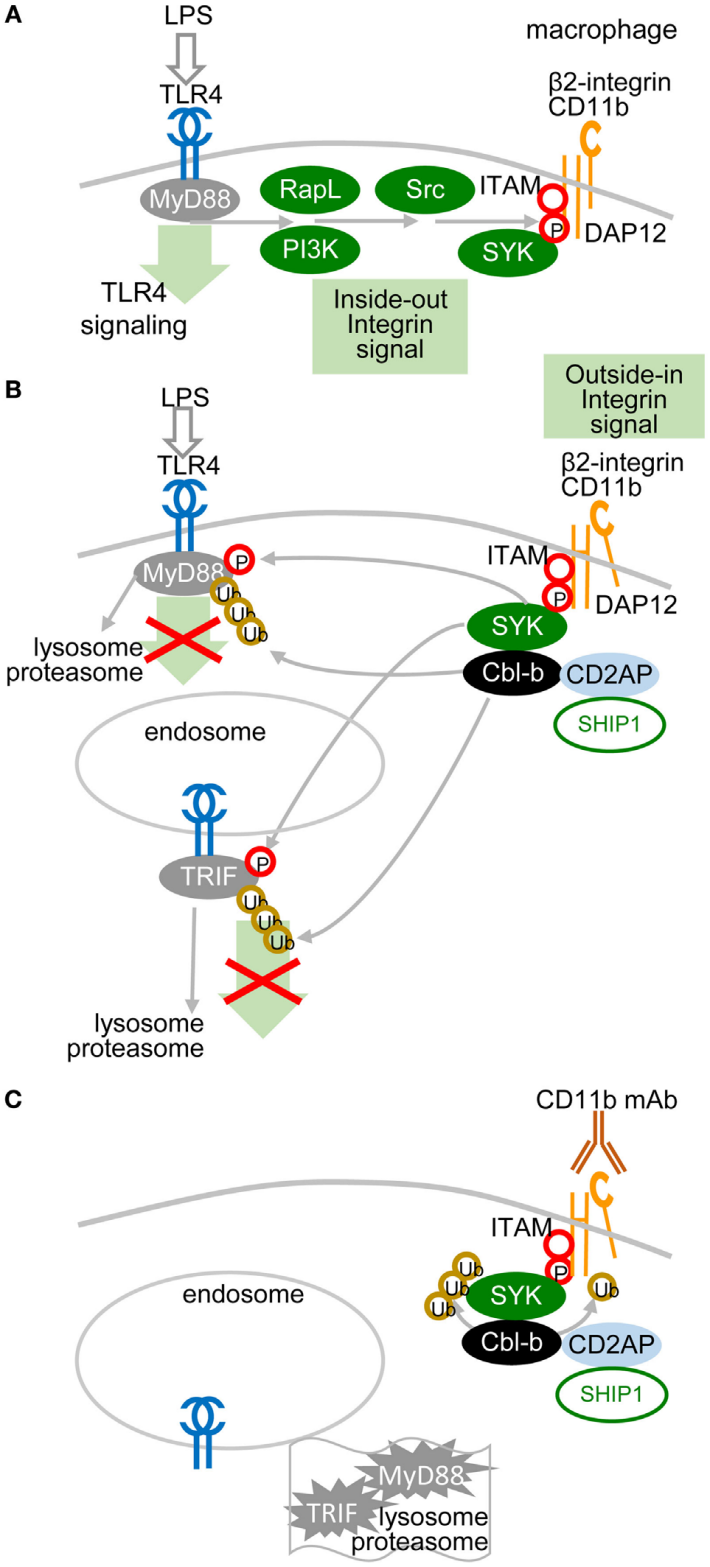
**Low-avidity signaling of β2-integrin regulates toll-like receptors (TLR) signaling in macrophages**. **(A)** Inside-out signals are initiated by TLR4 activation through phosphatidylinositol 3-OH kinase (PI3K) and effector RapL β2-integrin by separation of the cytoplasmic domains of α- and β-integrin chains. **(B)** β2-integrin signals in a low-avidity (without even ligand binding) outside-in manner through ITAM-associated activation of the spleen tyrosine kinase (SYK) pathway, which induces phosphorylation of Tyr227 on MyD88 and of Tyr375 on TRIF. TLR4 signaling is regulated by Cbl-b-mediated degradation of MyD88 and TRIF. Skewed position of β2-integrin indicates conformational changes after separation of the cytoplasmic domains α and β. **(C)** Cbl-b associates with CD2-associated protein (CD2AP, CIN85) and enhances the ubiquitination and degradation of SYK and FcεRIγ. Ubiquitinated MyD88 and TRIF are degraded by the proteasome.

A recent study showed that the TLR pathway in macrophages could be inhibited by another molecular mechanism, in which ITAM-associated low-avidity signaling inactivates MyD88 (49) (Figure 3). In this mechanism, SRC kinases-activated SYK phosphorylates Tyr227 on MyD88 and Tyr375 on TRIF, which function as substrates of the E3 ubiquitin ligase Cbl-b activated by β2-integrin CD11b (integrin α_M_, Mac1). The role of β2-integrin in this interplay depends on the orientation of outside-in and inside-out signals. In inside-out signaling, TLR4 activates β2-integrin through PI3K and effector RapL by phosphorylation of the β2-integrin DAP12 adaptor ITAM, which attracts SYK (Figure 3A) and leads to separation of the cytoplasmic domains α and β of β2-integrin (Figure 3B). Then, in outside-in signaling, the activated β2-integrin engaged or not with a low-avidity ligand feeds back to inhibit TLR4 signaling by activation of SYK-mediated phosphorylation of MyD88 and TRIF, which are subsequently ubiquitinated by Cbl-b and degraded (49). Several reports have shown that upon low-avidity β2-integrin stimulation, Cbl-b associates with CD2-associated protein (CIN85) and enhances the ubiquitination and degradation of SYK and FcεRIγ, resulting in inhibition of RRs (BCR-like) signaling (49–52) (Figure 3C). It has been reported that activation of SYK at the plasma membrane suppresses the TRAF6- and TAK1-mediated pro-inflammatory pathway and in contrast enhances the production of IFN-I *via* TBK-1 and IRF3 activation (53, 54). Thus, the global activation status of the target cell will be responsible for the outcome of TLR signaling.

In contrast to the well-established role of MAPK in activation of IFN-β production, several recent reports highlight suppressive aspects of MAPK signaling in myeloid cells (41, 43, 55–59). A central regulator that modulates repartition of MAPK signaling is Tumor Progression Lokus 2 (TPL-2 or Map3k8). TPL-2 downmodulates production of IFN-β and IL-12 (57) in macrophages, while it induces production of TNF-α (60) and IL-1β (61). It has been shown that activation of the MAPK (ERK) pathway by TPL-2 results, in macrophages but not in pDCs, in translocation of c-Fos into the nucleus and in inhibition of IFN-β gene transcription (57).

## High-Avidity Engagement of the Itam-Associated RRs in pDCs Inhibits TLR7/9 Signaling

**Figure 4:**
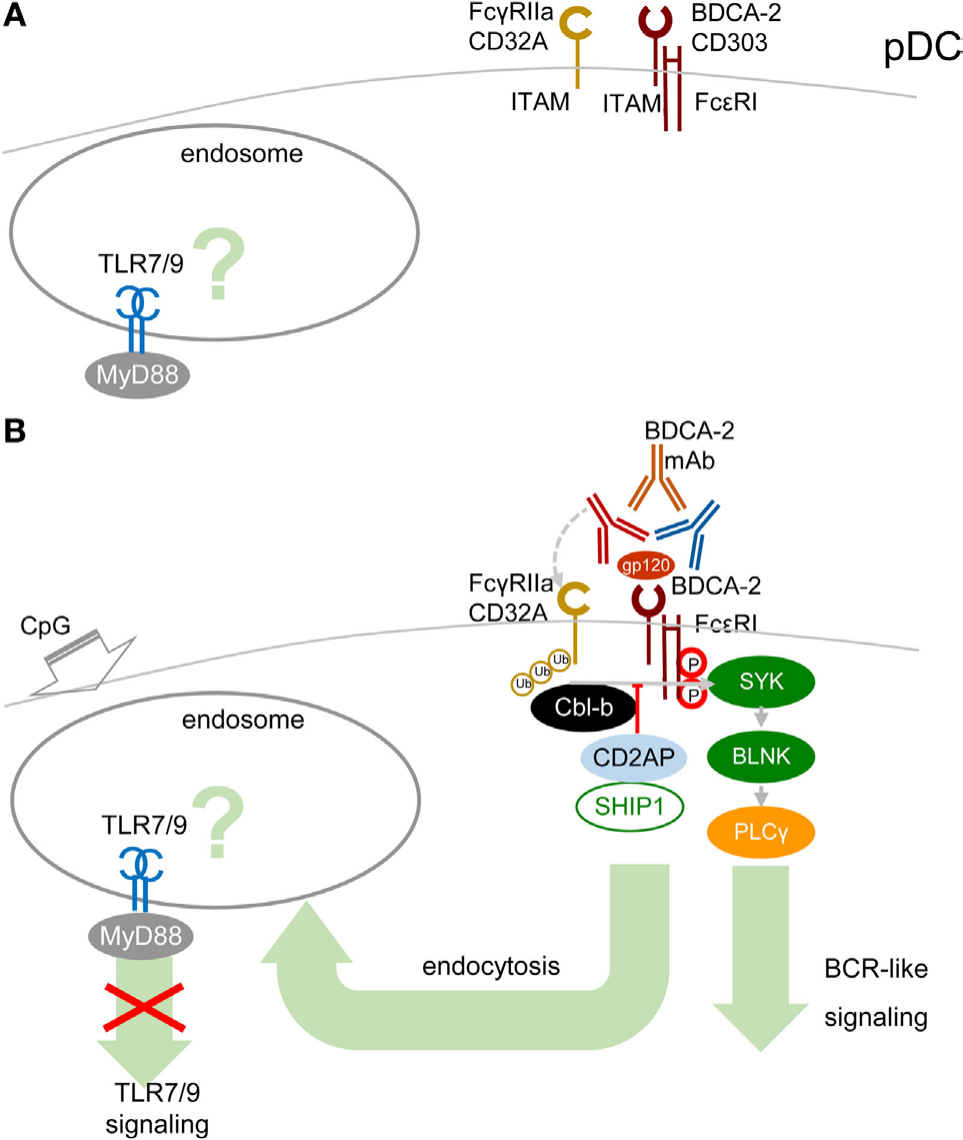
**High-avidity engagement of the immunoreceptor tyrosine-based activation motif (ITAM)-associated receptors in plasmacytoid DCs (pDCs) inhibits TLR7/9 signaling**. **(A)** In immature pDCs, blood dendritic cell antigen 2 (BDCA-2) and CD32a (FcγRIIa) are expressed on the cell surface. Homeostatic levels of TLR7/9 are present in endosomes. **(B)** BDCA-2 mAb or HIV-1 gp120/anti-gp120 Ab complex simultaneously engages BDCA-2 and CD32a, leading to internalization of both receptors. Crosslinking of BDCA-2-induces B-cell receptor (BCR)-like ITAM-mediated signaling. BDCA-2 cross-linking induces formation CD2AP/SHIP1 complex, which inhibits the Cbl-b-induced ubiquitination of spleen tyrosine kinase (SYK), FcεRIγ, and FcγRIIa and maintains the expression levels of SYK and FcεRIγ.

A cornerstone of the signal-switch hypothesis in macrophages is a direct relation between the avidity of the ITAM-associated receptor engagement and the intensity of IFN-I production (8, 9, 39, 45, 46). Surprisingly, in pDCs, the high-avidity cross-linking of BDCA-2 with mAb, as documented from protein tyrosine phosphorylation, activation of PLCγ, and intracellular Ca^2+^ release, results in the attenuation of TLR7/9-induced production of IFN-α and pro-inflammatory cytokines (17, 18) (Figure 4). As we and others demonstrated in pDCs, in contrast to macrophages, low-avidity engagement of BDCA-2 with monovalent anti-BDCA-2 Fab fragment, which does not induce any protein tyrosine phosphorylation in pDCs, fails to inhibit IFN-α production (62). As with BDCA-2, it is also the case that high-avidity engagement of the FcεRIγ_ITAM_-associated receptor ILT7 or FcεRIα, or of the DAP12_ITAM_-associated natural cytotoxicity receptor NKp44, is accompanied by protein tyrosine phosphorylation, calcium influx, and inhibition of IFN-I and pro-inflammatory cytokine production (19–22, 63). In addition to cross-linking with mAbs, high-avidity engagement of ILT7 with its natural ligand, bone marrow stromal cell antigen 2 (BST2, also called CD317, tetherin, or HM1.24) (19, 20, 63); of BDCA-2 with HIV-1 gp120 (64) or hepatitis C virus E2 glycoprotein (65); and of NKp44 with proliferating cell nuclear antigen also result in inhibition of IFN-I production (66).

The mechanism explaining inhibition of TLR7/9 signaling by high-avidity engagement of the ITAM-associated receptors in pDCs is not clear. The principal difference between pDCs and macrophages could reside in the localization and timing of the early steps of interaction of TLRs and ITAM-associated receptors. While in macrophages, the TLR4 and the ITAM-mediated β2-integrin signaling are concomitantly triggered from a close vicinity in the lipid raft (Figure 3), in pDCs, the TLR7/9 signaling is triggered from an endosome, whereas ITAM-mediated RRs signaling is triggered from the plasma membrane, with an unknown delay. Earlier findings suggested that in unstimulated pDCs, TLR7 and TLR9 reside in the endoplasmic reticulum and are delivered to the endolysosomal compartment only after uptake of RNA or DNA ligands to endosomes (10, 67, 68). However, more recent studies have demonstrated a steady-state flow of TLR9 from endoplasmic reticulum to endolysosomes, where TLR9 is present in the mature, cathepsin-cleaved form (69, 70) (Figure 4A). Most of these studies were performed in mouse bone marrow-derived macrophages, and parallel experiments in B cells showed that TLR9 trafficking is cell-context-dependent, making an actual localization of TLR7/9 in pDCs a matter of debate (69, 70). As with TLR9 trafficking, trafficking of RRs in pDCs remains elusive. Formation of the antibody–BDCA2 receptor complex (17), which colocalizes with EEA1 in early endosomes 5–10 min after crosslinking of BDCA2, was demonstrated in several laboratories (71, 72). Results from our laboratory have shown that 2 min after crosslinking of BDCA-2, SYK is phosphorylated, without assigning the phosphorylation to plasma membrane or endosome (73) (Figure 4B). Whether TLR7/9 and BDCA-2 co-localize in endolysosomes (71, 72), and at what level, remains to be determined.

As in macrophages, ubiquitination of MyD88, SYK, and FcεRIγ could play a crucial role in the outcome of TLR7/9 and RRs signaling in pDCs. Ubiquitination of these molecules depends on the cellular context. It has been shown that upon BDCA2 cross-linking in human pDCs, CD2AP forms a complex with SHIP1 and Cbl-b with reduced Cbl-E3 ubiquitin ligase activity in comparison with CD2AP- or SHIP1-knocked-down pDCs (3, 74) (Figure 4B). The CD2AP/SHIP1/Cbl complex is then recruited to the plasma membrane, where it co-localizes with cross-linked BDCA2/FcεR1γ complex. Inhibition of the Cbl-b-induced ubiquitination and degradation of FcεR1γ and SYK by the CD2AP/SHIP1 complex results in upregulation of BCR-like signaling and inhibition of TLR7/9 signaling. Although these results were not reproduced in mouse pDCs (75), differences between the triggering of TLR and ITAM signaling in macrophages and in pDCs (Figures 1 and 2) might be responsible for preferential ubiquitination of MyD88, SYK, and FcεRIγ in these cells.

In addition to ITAM-associated RRs, pDCs express an ITAM-associated FcR, FcγRIIa (CD32a), which is responsible for uptake and delivery of systemic lupus erythematosus (SLE) immune complexes (IC) in the endosomal compartment called the IFN signaling compartment, from where they trigger TLR9 signaling followed by massive IFN-I production (72, 76). ITAM-associated CD32a and ITIM-associated CD32b are the only FcRs on pDCs (72, 76). Surprisingly, BDCA-2 mAb simultaneously ligates BDCA-2 with F(ab′)_2_ and CD32a with the Fc region of the same single BDCA-2 mAb molecule, leading to the concurrent internalization of BDCA-2 and CD32a (Figure 4B). Simultaneous engagement of BDCA-2 and CD32a with complete BDCA-2 mAb, in contrast to ligation with F(ab′)_2_, synergizes inhibition of TLR9 signaling triggered by FcR-dependent SLE IC. However, simultaneous engagement does not potentiate inhibition of TLR7/9 signaling triggered by FcR-independent agonists CpG-A, CpG-B, and R848 (72). Thus, the latter results do not lend any support to the hypothesis that the simultaneous ligation of BDCA-2 and CD32a with a single mAb molecule, creating a trimolecular complex, would be responsible for BDCA-2 mAb-mediated inhibition of TLR9 signaling and IFN-I production (9, 38, 46, 47, 72). The synergistic effect of simultaneous engagement of CD32a and BDCA-2 highlights the therapeutic potential of these mAbs for inhibition of IFN-I production and for treatment of autoimmune diseases, such as SLE. Next, experiments can show whether natural ligands of RRs, such as BST2, HIV-1 gp120, hepatitis C virus E2 glycoprotein, or their complexes with antibody could cross-link BDCA-2/ILT7 and CD32a and inhibit TLR7/9 signaling (Figure 4B).

## Tolerogenic Effect of the High-Avidity Engagement of Itam-Associated Receptors in pDCs

Antigen targeted to pDCs by means of BDCA-2 mAb is rapidly endocytosed and traffics *via* early endosomes to MHC-enriched endosomes independently of TLR7/9 stimulation (71). However, the next steps of antigen presentation, including restimulation of antigen-specific CD4^+^ effector memory T helper cells, are dependent on TLR7/9 stimulation of pDCs, which is inhibited by BDCA-2 cross-linking. Mature pDCs are characterized by the upregulation of CD40 and co-stimulatory molecules, including CD80 and CD86. Recent results have shown that BDCA-2 mAb cross-linking inhibits CpG-A and CpG-B-induced upregulation of co-stimulatory molecules CD40 and CD86 (62, 77–79). In contrast, CD40L-stimulated upregulation of CD86 in pDCs is unaffected by BDCA-2 cross-linking. These results suggest that BDCA-2 signaling interferes with TLR9 signaling in pDCs but probably not with T-cell-dependent pDC activation *via* CD40-ligand (62). Actually, pDCs efficiently cross-present exogenous antigens to CD8^+^ T cells (80). Also, BDCA-2 agonist HIV-1 gp 120, but not the natural agonist of ILT7, BST2, suppressed TLR9-mediated expression of co-stimulatory molecules CD80 and CD86 by pDCs (19, 64).

The capacity of pDCs to produce IFN-I and their central role at the interface of innate and adaptive immunity could make them important actors in antitumor immunity (81). However, recent evidence suggests that tumor-associated (TA) pDCs recruited in breast and ovarian tumors are dysfunctional and their presence in these tumors is a negative prognostic factor for overall survival (82–84). This dysfunctionality is characterized by impairment of their IFN-I secretion and by strong expression of ICOS ligand, which leads to induction of immunosuppressive regulatory T cells (Treg) and priming of IL-10-secreting CD4^+^ T cells (82, 83). Apart from tumor-derived soluble immunosuppressive factors, such as TNF-α and TGF-β (85), recent data suggest that TA-pDC impairment could also result from the interaction of ITAM-associated RRs of pDCs with their ligands expressed on cancer cells, such as BST2 (20). Recent data from the C. Caux laboratory have shown that mAbs against ICOS inhibit TA-Treg expansion and IL-10 secretion, demonstrating a pivotal role of TA-pDCs in the immunosuppressive mechanism (82, 83). Collectively, these results indicate that a tumor microenvironment induces a tolerogenic character in pDCs.

## Concluding Remarks

**Figure 5:**
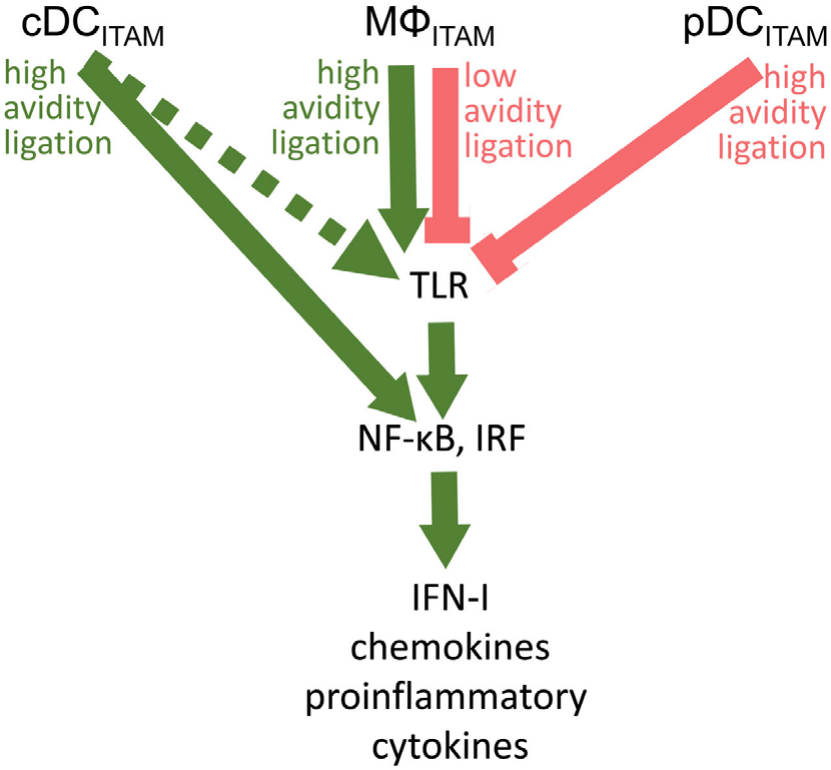
**Cross talk between immunoreceptor tyrosine-based activation motif (ITAM)-associated receptor signaling and toll-like receptor (TLR) pathways in conventional dendritic cells (cDCs), MΦ, and plasmacytoid DCs (pDCs): an ITAM-centric view**. ITAM-mediated activation pathways are shown by green arrows; ITAM-mediated inhibitory pathways are shown by red lines. Positive or negative control of immune responses in macrophages (MΦ) is determined by avidity of ITAM-associated receptors to their ligands. Production of interferons (IFNs)-I is facilitated by interferon-regulatory factor 3 (IRF3) in macrophages, by IRF5 in cDCs, and by IRF7 in pDCs. In cDCs, ITAM-associated receptor signaling can result in the IRF5-mediated production of IFN-β without engagement of TLRs (31). Alternative pathway in cDCs (32) is shown by dotted arrow.

Fifteen years after the discovery of the inhibitory role of BDCA-2 in IFN-I production in pDCs (17), its molecular mechanism remains elusive. The signal-switch hypothesis had a seminal role in the understanding of cross-regulation of cytokine- and TLR-signaling pathways in macrophages (8, 9, 28, 39, 58). However, further studies showed that the ITAM-signaling pathway may be regulated in a special way in human pDCs (11, 18–20). While the high-avidity engagement of ITAM-associated receptors in macrophages leads to potentiation of TLR signaling, it results in the attenuation of TLR-induced IFN-I production in pDCs (Figure 5; Table 1). Surprisingly, few data are available on the interplay of ITAM-associated receptors and TLRs in cDCs. Cellular context, spatiotemporal differences, and different functions of ITAM-associated receptors could be responsible for the different interplay of ITAM and TLR pathways in pDCs, cDCs, and macrophages.

**Table 1:** **Cross talk between immunoreceptor tyrosine-based activation motif (ITAM)-signaling and toll-like receptors (TLR) pathways in macrophages and plasmacytoid DCs (pDCs)**.^a^

	TLR		ITAM-coupled receptors				ITAM/TLR cross talk	
	Receptor	Ligand	Receptor	Adaptor	Ligand		High-avidity ligation	Low-avidity ligation
					High avidity	Low avidity		
Macrophage	TLR4	LPS	β_2_-Integrin FcγRI FcγRIIA FcαR FcεRI TREM2	DAP12 FcRγ – FcRγ FcRγ DAP12	Fibrinogen IC/RF IC/RF IC/RF IC/RF poly RGD	ECM Monomeric IgG or IgA, IVIg, mAb F(ab′)_2_ Semaphorin 6D	Synergizes TLR signaling Inhibits cytokine signaling Activation	Inhibits TLR signaling Enhances cytokine signaling Homeostasis

pDCs	TLR7	ssRNA Resiquimod	BDCA-2	FcεR1γ	HIV gp120, HCV E2; mAb	mAb Fab, F(ab′)_2_	Inhibits TLR signaling Inhibits pDC maturation and T cell stimulation Homeostasis/anergy	No/unknown effect
			ILT7	FcεR1γ	BST2			
			FcεRIα	FcεR1γ	IgE			
			NKp44	DAP12	PCNA			
	TLR9	CpG ODNs	Siglec-H	DAP12	Sialic acid			
			FcγRIIA	–	IC			

*^a^mAb, mAb Fab, and mAb F(ab′)_2_ are related to the respective receptor*.

*IC, immune complexes; RF, rheumatoid factor; IVIg, intravenous immunoglobulin; TREM2, triggering receptor expressed on myeloid cells 2; ECM, extracellular matrix; RGD, arginine–glycyl–aspartic acid motif; DAP12, DNAX activation protein 12; BDCA-2, blood dendritic cell antigen 2; ILT7, immunoglobulin-like transcript; PCNA, proliferating cell nuclear antigen*.

Published data suggest that ITAM-associated receptors play different roles in pDCs, cDCs, and in macrophages. Under home-ostatic conditions in macrophages, the ITAM-associated receptors enable a fine-tuning of immune responses, including inhibition of IFN-I production and high sensitivity to extracellular cytokines. In an infection setting, ITAM-associated receptors in macrophages switch to signaling for robust production of cytokines including IFN-I, to cell activation and to low sensitivity to extracellular cytokines. In cDCs, ligation of ITAM-associated receptors leads to rapid activation of NF-ĸB and massive production of cytokines, which can occur without engagement of TLR. In contrast, the major role of ITAM-associated RRs in pDCs is related to inhibition of cytokine production and reestablishment of a tolerogenic state following pDC activation. Limitation of the ITAM-associated RR signaling in pDCs to high-avidity engagement could be related to a low homeostatic level of TLR7/9 in endosomes in immature pDCs under physiological conditions (10, 70). The low homeostatic level of TLR7/9 in endosomes in immature pDCs could reduce the risk of undesirable triggering of IFN-I signaling to the same extent as that of the inhibition of IFN-I induced in macrophages by tonic ITAM signaling. Also, simultaneous engagement of BDCA-2 and CD32a leading to the internalization of both receptors is consistent with the tolerogenic role of BDCA-2. Differential effects of ITAM-mediated signaling in pDCs and macrophages would promote a coordinated cellular response to infection and inflammation.

Interaction of the TLR pathway and ITAM signaling in pDCs plays an important role in control of the innate immune responses in viral infections (1, 64, 65), cancer proliferation (20, 83, 84), and autoimmune diseases (54, 86). Understanding the ITAM/TLR-signaling network in pDCs may serve as an effective means for positive and negative control of pDC activation. Progress in understanding these interactions paves the way for the development of compounds to control activation of pDCs. Pharmacologic targeting of TLR and ITAM signaling is thus an attractive new therapeutic approach for treatment of chronic infections, cancer, and autoimmune and inflammatory diseases.

## Author Contributions

The work was written by IH and NB-V with substantial contributions of VJ and RS to the conception, drafting, and revising the work for important intellectual content. All authors gave final approval of the version to be published; and agreement to be accountable for all aspects of the work in ensuring that questions related to the accuracy or integrity of any part of the work are appropriately investigated and resolved.

## Conflict of Interest Statement

The authors declare that the research was conducted in the absence of any commercial or financial relationships that could be construed as a potential conflict of interest. The reviewer, SM, and handling Editor declared their shared affiliation, and the handling Editor states that the process nevertheless met the standards of a fair and objective review.

## Abbreviations

BCR, B-cell receptor; BDCA-2, blood dendritic cell antigen 2; BLNK, B-cell linker protein; BTK, Bruton’s tyrosine kinase; CD2AP, CD2-associated protein (AP); cDC, conventional dendritic cell; DAG, diacylglycerol; DAP12, DNAX activation protein 12; DC, dendritic cell; DCIR, dendritic cell immunoreceptor; ECM, extracellular matrix; FcRs, Fc receptors; IC, immune complexes; IL-6, interleukin-6; IFN, interferon; ILT7, immunoglobulin-like transcript; IRAK1/4, interleukin-1 receptor-associated kinase 1/4; IRF7, interferon-regulatory factor 7; ITAM, immunoreceptor tyrosine-based activation motif; ITIM, immunoreceptor tyrosine-based inhibition motif; LLR, lectin-like receptors; MDL-1, myeloid DAP12-associated lectin-1; MICL, myeloid C-type lectin-like receptor; Mincle, macrophage-inducible C-type lectin; MAPK, mitogen-activated protein kinases; NF-κB, nuclear factor kappa B; pDC, plasmacytoid DC; PI3K, phosphatidylinositol 3-OH kinase; PLCγ2, phospholipase Cγ 2; PRRs, pathogen recognition receptors; RF, rheumatoid factor; RGD, arginine–glycyl–aspartic acid motif; RRs, regulatory receptors; SHIP, SH2-domain-containing inositol phosphatase-1; R848, Resiquimod; SHP1, SRC-homology-2 (SH2)-domain-containing protein tyrosine phosphatase 1; TAK1, transforming growth factor β-activated kinase 1; TCR, T-cell receptor; TLRs, toll-like receptors; TNF-α, tumor necrosis factor-α; Treg, regulatory T cells T cells; TRAF3/6, tumor necrosis factor receptor-associated factors 3 and 6; TREM2, triggering receptor expressed on myeloid cells 2; SYK, spleen tyrosine kinase.
